# Incidence, antimicrobial prescribing practice, and associated healthcare costs of paediatric otorrhoea in primary care in the UK: a longitudinal population study

**DOI:** 10.3399/BJGP.2024.0053

**Published:** 2024-12-10

**Authors:** Elliot Heward, Eleni Domzaridou, Sean P Gavan, Matthew Carr, Judith Lunn, John Molloy, Rachel Isba, Alastair D Hay, Jaya R Nichani, Iain A Bruce, Darren M Ashcroft

**Affiliations:** Division of Infection, Immunity and Respiratory Medicine, School of Biological Sciences, Faculty of Biology, Medicine and Health, University of Manchester, Manchester, and Royal Manchester Children’s Hospital, Manchester University Hospitals NHS Foundation Trust, Manchester.; Division of Pharmacy & Optometry, School of Health Sciences, Faculty of Biology, Medicine and Health, University of Manchester, Manchester, and NIHR Greater Manchester Patient Safety Research Collaboration (PSRC), University of Manchester, Manchester.; Manchester Centre for Health Economics, Division of Population Health, Health Services Research and Primary Care, School of Health Sciences, Faculty of Biology, Medicine and Health, University of Manchester, Manchester.; Division of Pharmacy & Optometry, School of Health Sciences, Faculty of Biology, Medicine and Health, University of Manchester, Manchester.; Lancaster Medical School, Lancaster University, Health Innovation One, Lancaster.; Division of Infection, Immunity and Respiratory Medicine, School of Biological Sciences, Faculty of Biology, Medicine and Health, University of Manchester, Manchester, and Royal Manchester Children’s Hospital, Manchester University Hospitals NHS Foundation Trust, Manchester.; Lancaster Medical School, Lancaster University, Health Innovation One, Lancaster, and Alder Hey Children’s Hospital, Alder Hey Children’s NHS Foundation Trust, Liverpool.; Centre for Academic Primary Care, Bristol Medical School: Population Health Sciences, University of Bristol, Bristol.; Division of Infection, Immunity and Respiratory Medicine, School of Biological Sciences, Faculty of Biology, Medicine and Health, University of Manchester, Manchester, and Royal Manchester Children’s Hospital, Manchester University Hospitals NHS Foundation Trust, Manchester.; Division of Infection, Immunity and Respiratory Medicine, School of Biological Sciences, Faculty of Biology, Medicine and Health, University of Manchester, Manchester, and Royal Manchester Children’s Hospital, Manchester University Hospitals NHS Foundation Trust, Manchester.; Division of Pharmacy & Optometry, School of Health Sciences, Faculty of Biology, Medicine and Health, University of Manchester, Manchester, and NIHR Greater Manchester Patient Safety Research Collaboration (PSRC), University of Manchester, Manchester.

**Keywords:** antibiotic resistance, incidence, otitis media, suppurative primary health care

## Abstract

**Background:**

Paediatric otorrhoea (PO) is a symptom-based diagnosis encompassing acute and chronic ear infections that cause otorrhoea in children and young people (CYP).

**Aim:**

To understand the burden of PO on primary care services.

**Design and setting:**

This was a longitudinal population study in UK primary care.

**Method:**

Data from the Clinical Practice Research Datalink (CPRD Aurum), January 2005 to December 2019, was analysed. CYP <17 years of age with otorrhoea were included. Standardised annual incidence and presentation rates were estimated. Poisson regression modelling was used to determine risk ratios comparing sex, age, and Index of Multiple Deprivation (IMD). A probabilistic simulation scaled-up estimates for the UK population.

**Results:**

The cohort included 6 605 193 CYP, observed over 32 942 594 person-years. There were 80 454 people with incident cases and 106 318 presentations of PO during the 15-year period, equating to standardised annual incidence and presentation rates per 1000 patient-years of 2.42 (95% confidence interval [CI] = 2.40 to 2.44) and 3.15 (95% CI 3.13 to 3.17), respectively. In the UK this equates to 41 141 primary care appointments per year. Incidence was higher in males, those aged 0–2 years, and those living in the least deprived quintile. Treatment involved oral antibiotics (57.1%, 45 931/80 454), no prescription (28.1%, 22 569/80 454), topical antibiotics (9.7%, 7797/80 545), or a combination (4.9%, 3910/80 545). The cost to NHS primary care is estimated at £1.97 million per year.

**Conclusion:**

To the authors’ knowledge, this is the first longitudinal population-based study investigating PO that demonstrates the burden on primary care. Antimicrobial prescribing predominantly follows National Institute for Health and Care Excellence guidelines using oral amoxicillin. Aminoglycosides are the most frequently prescribed topical antibiotic despite the concern of ototoxicity.

## Introduction

Paediatric otorrhoea (PO) starts with an infection behind the ear drum called acute otitis media (AOM). Pus in the middle ear space accumulates that can then cause the tympanic membrane to stretch until it ruptures, resulting in discharge. Most frequently the acute discharge resolves and the tympanic membrane heals. In some cases, inadequately treated middle ear infections lead to chronic foul-smelling drainage from the ear. In routine clinical practice, the presence, or absence, of otorrhoea is considered of greater relevance to treatment decisions than the chronicity of the infection. PO is a symptom-based diagnosis that represents real-world practice and encompasses acute and chronic middle ear infections in children and young people (CYP).

The incidence rate of paediatric AOM has previously been estimated to be 198–256 per 1000 person-years, with approximately 7–15% of AOM cases in patients resulting in otorrhoea.^1^^–^^4^ Hullegie *et al* reported the incidence of presentations with ear discharge in CYP aged 0–12 years over a 1-year period as 12.6 per 1000 patient-years.^5^ The prevalence of chronically discharging ears in CYP is variable. It is estimated that in developed and developing countries the incidence is <0.5 and 10–80 per 1000 people respectively.^6^

PO can lead to significant temporary or permanent morbidity in children, most frequently resulting in hearing loss, which may then lead to developmental delay, restricted communication, poor psychosocial development, and reduced educational attainment.^6^ Approximately 10% of CYP with persistent otorrhoea will develop permanent sensorineural hearing loss.^7^ There is also a significant impact on CYP (and their families), who experience offensive smelling discharge that can have a negative impact on education, sporting activities, and socialisation.^8^

**Table:** How this fits in

The burden of paediatric otorrhoea (PO) on primary care services in the UK has not, to the authors’ knowledge, previously been reported. This study shows the incidence and presentation rate of PO is 2.42 and 3.15 per 1000 patient-years, respectively. The incidence of PO is higher in males, those aged 0–2 years, and those living in the least deprived quintile. Oral antibiotics are the most frequent treatment (57.1%) for PO and the total cost to NHS primary care is estimated at £1.97 million per year.

Current National Institute for Health and Care Excellence (NICE) guidelines outline that CYP with AOM-related otorrhoea are more likely to benefit from antibiotic treatment in the form of oral amoxicillin.^9^ A meta-analysis has shown that pain and fever is significantly reduced in CYP who have otorrhoea that is treated with amoxicillin compared with placebo.^4^ Previous research has reported that 92% of CYP with acute otorrhoea receive antibiotics in primary care.^1^ The World Health Organization (WHO) recommends collecting data on antimicrobial prescribing to help improve antimicrobial stewardship and prevent resistance.^10^

The alternative treatment for PO is with topical antibiotics that can be used when a tympanic membrane perforation is present. The benefit of topical antibiotics, in comparison with oral antibiotics, is reduced systemic side effects such as nausea and diarrhoea.^9^ Aminoglycoside-containing topical antibiotics have potential ototoxic effects when used with a tympanic membrane perforation, but this has not been established with clear evidence.^11^ ENT UK has recommended using these drops only with clear justification. Alternative, non-ototoxic drops such as ciprofloxacin have been recommended off-licence.^11^

To enable delivery of healthcare provision and future high-quality research, the current study aimed to determine the incidence of PO in the UK, understand current treatment practice in primary care, and estimate associated healthcare costs.

## Method

### Study design and setting

The study protocol was registered (ISCTRN43760) and published online.^8^ Approval was granted by the Clinical Practice Research Datalink (CPRD) research and governance process on 4 April 2023 (22_002508). Anonymised primary care electronic health records from the CPRD Aurum database were used.

The CPRD Aurum database collects fully coded electronic patient records from GP in the UK using EMIS® software. As of June 2021, CPRD Aurum included 1491 (21%) GP practices from England and Northern Ireland. It has been found to be representative in terms of age, sex, and ethnicity compared with the UK population.^12^^,^^13^

The study cohort was linked to practice-level Index of Multiple Deprivation (IMD) quintiles that were derived from information on relative deprivation. IMD is measured across 32 844 small geographic areas or neighbourhoods that include on average 1600 residents and are known as lower-level super output areas.^14^ The IMD score is derived from variables that reflect variability in factors such as income, employment, education, health, crime, living environment, and access to services. Sex assigned at birth recorded on the electronic health records was used.

Patients ≤16 years of age registered with a GP within the observation period (1 January 2005 to 31 December 2019) were included. The observation period was selected to avoid the effects of the COVID-19 pandemic. Patients were observed until the earliest occurrence of: last date of data collection (the last date a GP contributed data to the CPRD), transfer out of practice date, 16th birthday, death, or the end of the study’s observation period. Supplementary Figure S1 illustrates the study design. The analysis was restricted to include patients with acceptable-quality data for research according to CPRD quality standards. Supplementary Figure S2 shows the steps of data extraction.

### Outcomes and clinical codes

The primary outcome was the calculation of incidence rates of PO diagnoses. PO diagnoses were identified using medical code lists of Read, SNOMED, or EMIS codes in CPRD Aurum (Supplementary Table S1). The clinical code lists were reviewed by three otolaryngology clinicians and one GP clinician (the first, eight, ninth, and tenth authors). For codes to be included, all clinicians had to be in consensus. The secondary outcome was the calculation of presentation rates (repeated PO diagnoses per patient) of PO. To calculate presentation rates, a 6-week window was applied between repeated events to allow time for recovery and avoid double counting the same clinical events. Patients’ exposure to antibiotic and antifungal treatments that were prescribed at PO diagnosis date were also examined, excluding duplicated prescriptions (same product code) issued at the same date.

### Frequency measures

The incidence of PO was defined as the patient’s first episode of PO within the study period. Presentations were defined as the total number of separate attendances with PO episodes (including the first incident case). A minimum of 6 weeks between episodes was used to allow for infection resolution between each episode.

Overall and annual incidence and presentation rates were examined and compared between sexes, and among age bands, and IMD quintiles. For new cases, patients’ follow-up time was censored to their first recorded PO event, whereas for repeated events all the available follow-up time was considered.

### Statistical analyses

To allow comparability between groups, all rates were directly standardised using the CPRD population, and calculated by dividing the number of PO diagnoses with the number of person-years of follow-up among patients of the same sex, age band, IMD quintile, and GP geographical region. The rates were reported per 1000 person-years of follow-up. Person-years take into account the number of observed people multiplied by the number of years of observation.

Risk ratios (RRs) with their 95% confidence intervals (CIs) were estimated by applying Poisson regression whereby the number of PO diagnoses was the outcome, and the logarithm of the total person-years at risk was used as an offset. The models were adjusted for age, sex, IMD quintiles, geographical region, and calendar year as a categorical variable to account for potential variability of the risk over time. There was <1% missing data for IMD; these patients were removed before modelling. There were no other missing data. Robust standard errors were used for the parameter estimates to account for potential violation of the assumption that the variance equals the mean. All the analyses were conducted using R version 4.0.2.

### Population impact

The outputs from the CPRD analysis were scaled up to the UK population using a probabilistic simulation. The total number of people aged between 0 and 16 years (inclusive) in the UK were obtained from Office for National Statistics data.^15^ The incidence of presentations was sampled from a gamma distribution. Dirichlet distributions were used to sample:

- the probability of receiving treatment (oral antibiotic, topical antibiotic, antifungal, or no treatment); and
- the specific treatment received based on the frequency of these prescriptions in the CPRD output.

The unit cost of primary care visits was obtained from the *Unit Costs of Health and Social Care 2022* manual.^16^ The unit costs of prescribed items were obtained from the *British National Formulary for Children*.^17^

The results reported the estimated annual population-level quantity of primary care visits, items prescribed, and direct healthcare cost to primary care of PO in the UK. Costs were measured from the perspective of the healthcare system (price year: 2022/2023). Monte Carlo simulation sampled plausible values for the incidence and probability parameters from their respective distributions at random (*n* = 10 000 simulations). Results were calculated as the expected (mean) values over these simulations with 95% CIs. All simulation analyses were performed in Microsoft Excel (version: 2309).

### Patient and public involvement

Patients and the public helped shape the design of this study. A meeting with young service users, on 22 November 2023, helped interpret the study findings outlined in this manuscript.

## Results

### Cohort characteristics

The study cohort included 6 605 193 CYP, observed over 32 942 594 person-years, from 1491 general practices. Analysis identified 80 454 diagnoses of PO during the observation period. Among patients with a PO diagnosis, 54.1% (43 559/80 454) were male, 35.5% (28 584/80 545) were aged ≤2 years, and 23.4% (18 847/80 454) lived within neighbourhoods in the most deprived quintile (Supplementary Table S2). In total, 80.6% (64 846/80 454) of CYP were diagnosed with PO once and 19.4% (15 608/80 454) more than once (median: 2; interquartile range: 1–3). Of the CYP who re-presented with PO within 3 months of their first diagnosis, 74% (4352) had been treated with antibiotics at their first attendance.

Descriptive analysis that accounted for job roles showed that 82.9% (47 991/57 887) of prescribers were GPs and 13.7% (7920/57 887) were other healthcare professionals (the remaining 3.4% [1976/57 887] were missing data) (Supplementary Table S3).

### Overall standardised rates and adjusted analyses

The standardised annual incidence and presentation rates were 2.42 (95% CI = 2.40 to 2.44) and 3.15 (95% CI = 3.13 to 3.17) per 1000 person-years, respectively (Table 1). Findings from the Poisson regression models showed that, compared with males, female patients had a lower rate of PO diagnosis (RR 0.89, 95% CI = 0.88 to 0.91). Compared with patients aged <2 years of age, older children had a decreasing incidence: aged 2–5 (0.41, 95% CI = 0.40 to 0.42), 5–9 (0.24, 95% CI = 0.23 to 0.24), or 8–16 years (0.09, 95% CI = 0.09 to 0.09).

**Table 1.** Crude, standardised, and adjusted incidence and presentation rates by sex, age, and deprivation quintile between 2005 and 2019

**Characteristic**	**Diagnoses**	**Person-years**	**Crude rate^a^**	**Standardised rates**	**Risk ratio (95% CI)^b^**
**Incidence of PO**	80 454	32 942 594	2.44	2.42	CI: 2.40 to 2.44
Sex					
Male	43 559	17 047 829	2.56	2.55	1 (Reference)
Female	36 895	15 894 765	2.32	2.29	0.89 (0.88 to 0.91)
Age band, years					
0–2	28 584	3 199 849	8.93	9.07	1 (Reference)
3–5	24 271	6 639 917	3.66	3.68	0.41 (0.40 to 0.42)
6–8	13 849	6 403 053	2.16	2.16	0.24 (0.23 to 0.24)
9–16	13 750	16 699 775	0.82	0.82	0.09 (0.09 to 0.09)
IMD quintiles^c^					
1 (least deprived)	14 972	5 298 858	2.83	2.55	1 (Reference)
2	14 179	5 013 187	2.83	2.81	1.00 (0.98 to 1.03)
3	16 273	6 538 914	2.49	2.45	0.87 (0.85 to 0.89)
4	15 724	7 298 310	2.15	2.22	0.74 (0.72 to 0.76)
5 (most deprived)	18 847	8 578 013	2.20	2.26	0.77 (0.75 to 0.78)

**Presentations of PO**	106 318	33 382 591	3.18	3.15	CI: 3.13 to 3.17
Sex					
Male	57 803	17 287 760	3.34	3.33	1 (Reference)
Female	48 515	16 094 831	3.01	2.96	0.89 (0.88 to 0.90)
Age band, year					
0–2	41 698	5 440 221	7.66	7.78	1 (Reference)
3–5	30 128	6 690 322	4.50	4.52	0.58 (0.56 to 0.60)
6–8	17 484	6 424 477	2.72	2.72	0.36 (0.35 to 0.36)
9–16	17 008	14 827 571	1.15	1.14	0.15 (0.15 to 0.15)
IMD quintiles^c^					
1 (least deprived)	20 105	5 384 216	3.73	3.64	1 (Reference)
2	18 835	5 093 609	3.70	3.97	0.99 (0.97 to 1.01)
3	21 654	6 628 570	3.27	3.50	0.87 (0.85 to 0.89)
4	20 416	7 381 390	2.77	3.11	0.73 (0.71 to 0.74)
5 (most deprived)	24 686	8 676 445	2.85	3.23	0.75 (0.69 to 0.82)

a *Calculated per 1000 person-years of follow-up.*

b *Risk ratios were adjusted for geographical region and calendar year.*

c *IMD data were missing for 458 patients at first presentation (incidence) and for 622 presentation episodes. CI = confidence intervals. IMD = Index of Multiple Deprivation. PO = paediatric otorrhoea.*

Patients who lived in neighbourhoods in the least deprived areas had higher rates of PO diagnosis compared with patients who lived in neighbourhoods in the remaining IMD quintiles (second to fifth quintiles). The presentation rates analysis yielded similar findings (Table 1).

### Annual standardised rates and findings from stratified analyses

**Figure 1.**
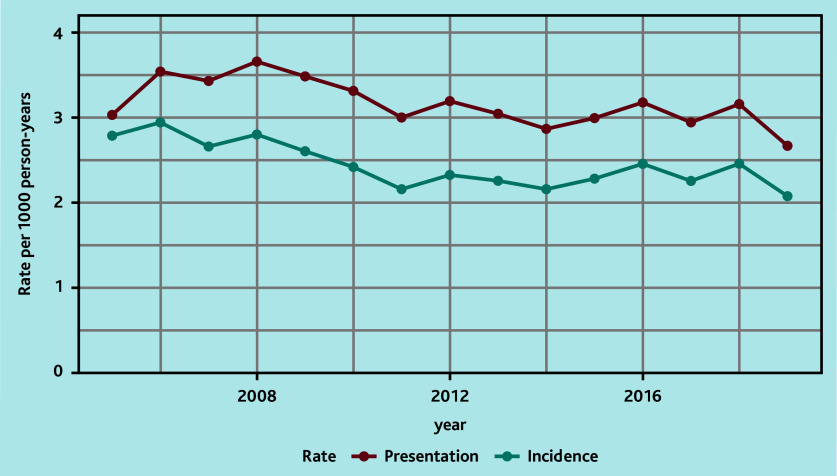
Overall standardised annual incidence and presentation rates.

**Figure 2.**
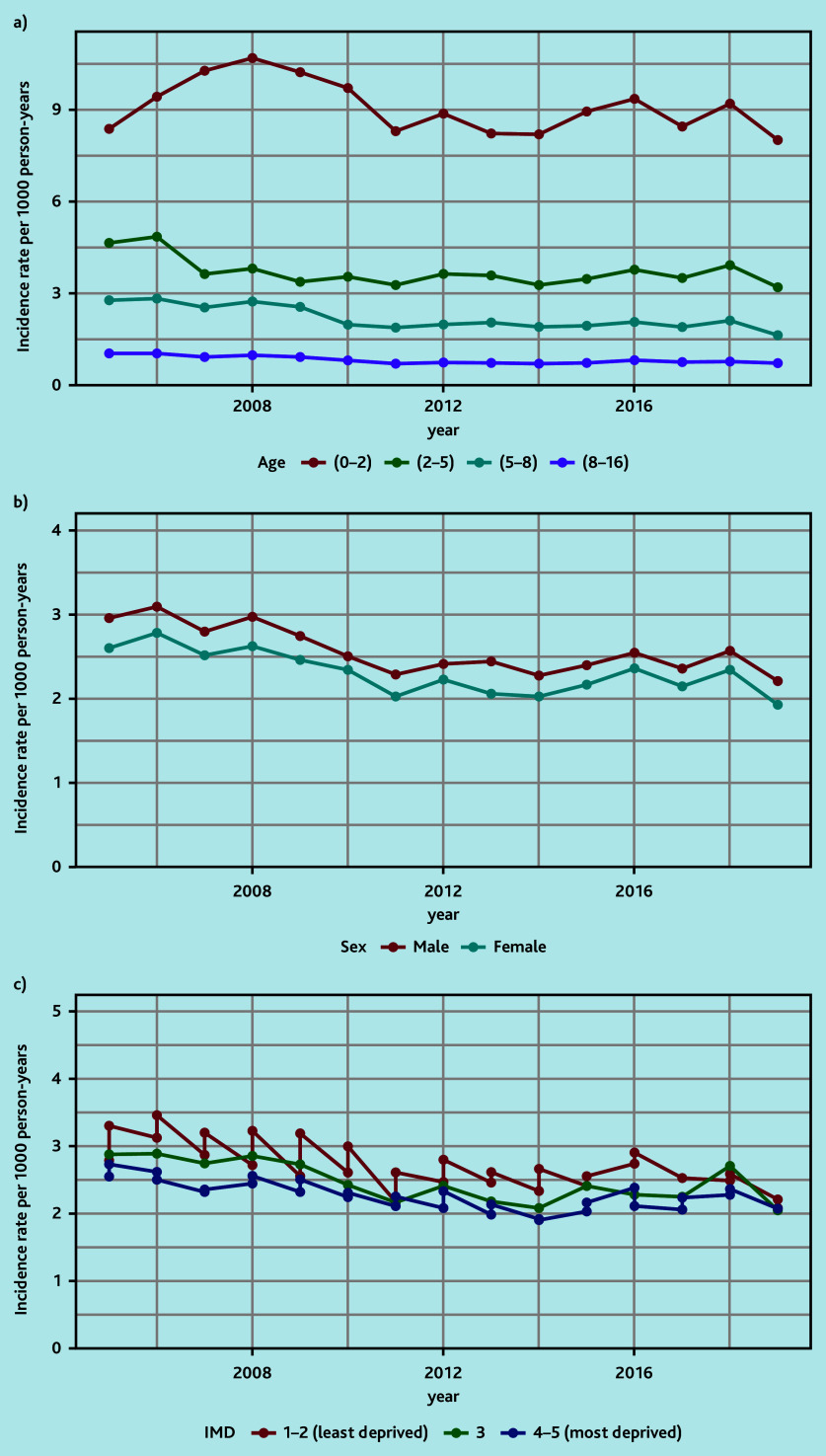
Annual standardised incidence rates by a) age (years), b) sex, and c) deprivation quintile. IMD = Index of Multiple Deprivation.

The annual standardised incidence rates over time showed decreasing rates during 2008–2011 and a stable rate during 2011–2019 (Figure 1, Supplementary Table S4). The corresponding presentation rates showed a similar pattern, with rates fluctuating between 2.7 and 3.7 per year (Figure 1, Supplementary Table S5). This pattern was also demonstrated when stratifying patients by age, sex, and deprivation (Figure 2, Supplementary Tables S6–S11).

### Antibiotic and antifungal exposure

In terms of treatments prescribed at the time of diagnosis, 71.9% (57 885/80 454) of patients were prescribed antibiotics or antifungal drugs and 28.1% (22 569/80 454) of patients received no prescription. The majority (79.3%, 45 931/57 885) of patients who received antibiotics or antifungal treatment were prescribed exclusively oral antibiotics, 13.5% (7797/57 885) only topical antibiotics, and 6.8% (3910/57 885) were prescribed both formulations. The study found that <1% (247/57 885) of patients were prescribed oral or topical antifungal drugs alone. Antifungals were prescribed in combination with antibiotics in 555 patients.

**Figure 3.**
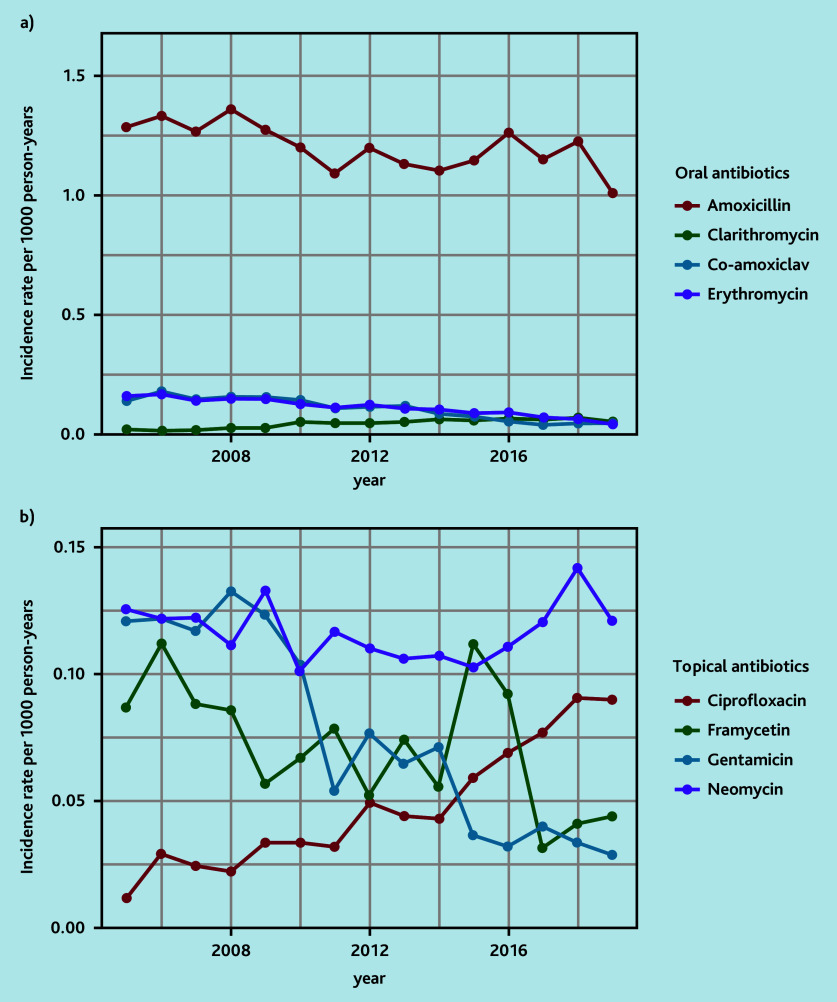
Annual rates of a) oral and b) topical antibiotic prescriptions.

Amoxicillin and neomycin were the most frequently prescribed oral and topical antibiotics, respectively (Table 2). Prescriptions of aminoglycoside-containing topical drops have fallen and ciprofloxacin topical drops have increased over time (Figure 3, Supplementary Tables S12 and S13). The antibiotic prescription rate was similar in all IMD groups (IMD 1: 71.9% [10 766/14 972], IMD 2: 71.7% [10 172/14 179], IMD 3: 71.0% [11 550/16 273], IMD 4 7: 1.7% [11 270/15 724], IMD 5: 71.8% [13 528/18 847]).

**Table 2.** Frequency of prescribed antibiotics at first attendance with paediatric otorrhoea^a,b^

**Formulation and substance**	**Patients, *n***	**Value, %**
**Oral**	50 067	
Amoxicillin	39 431	79.1
Erythromycin	3661	7.3
Co-amoxiclav	3434	6.9
Clarithromycin	1592	3.2
Cefalexin	723	1.4
Phenoxymethylpenicillin	286	0.6
Cefaclor	268	0.5
Trimethoprim	248	0.5
Azithromycin	157	0.3
Ciprofloxacin	132	0.3
Ampicillin	32	<0.1
Cefixime	28	<0.1
Cefuroxime	23	<0.1
Cefadroxil	17	<0.1
Other	35	<0.1

**Topical**	12 122	
Neomycin	3847	31.7
Gentamicin	2415	19.9
Framycetin	2320	19.1
Ciprofloxacin	1637	13.5
Chloramphenicol	890	7.3
Fusidic acid	874	7.2
Mupirocin	66	0.5
Clindamycin	24	0.2
Oxytetracycline	20	0.2
Erythromycin	15	0.1
Other	14	0.1

a *The sum inTable 2 includes cases where patients received more than one antibiotic prescription on the same day, leading to non-mutually exclusive counts in the cells.*

b *Aminoglycosides include: neomycin, gentamicin, and framycetin.*

### Population impact

By scaling-up to the UK population-level, 41 141 primary care presentations were estimated per year, resulting in 31 887 items prescribed annually. The total expected cost of PO to NHS primary care services is £1 971 680 per year (Table 3).

**Table 3.** Population-level results^a^

**Population-level result**	**Value (95% CI)**
**Number of presentations per year**	41 141 (41 116 to 41 167)
**Total cost of presentations per year, £**	1 686 790 (1 685 754 to 1 687 827)
**Number of prescriptions per year**	31 887 (31 867 to 31 906)
**Total cost of prescriptions per year, £**	284 890 (284 714 to 285 066)
**Total cost to primary care per year, £**	1 971 680 (1 970 469 to 1 972 891)

a *Price year: 2022/2023. CI = confidence interval.*

## Discussion

### Summary

This is, to the authors’ knowledge, the first longitudinal population-based study to describe the incidence of new presentations of PO to primary care. The results demonstrate that in the UK approximately 31 650 CYP present to NHS primary care services with new otorrhoea; including re-attendances a total of 41 141 primary care appointments occur.

Antibiotics are prescribed to 71.9% (57 885/80 454) of CYP with PO, of which 79.3% (45 931/57 885) received oral antibiotics. The majority of oral antibiotic prescriptions (amoxicillin or clarithromycin: 89.3% [41 023/45 931]) follow NICE guidelines.^9^

### Strengths and limitations

The main limitation of this study relates to coding within the CPRD database. Diagnoses cannot be captured by the CPRD if individuals do not present to health services or if they were recorded using free-text entries. In addition, otorrhoea is a complication of AOM; coding the episode as AOM would not be captured in the results. It is expected this work underestimates the exact disease burden.

Otorrhoea in the paediatric population most frequently presents because of AOM. It can also, less frequently, be caused by otitis externa in teenagers. The authors have taken a pragmatic approach by collecting both diagnostic codes and clinical signs (for example, otorrhoea), which inevitably will identify a number of CYP with otitis externa. In this study, conditions causing otorrhoea have been grouped as ‘paediatric otorrhoea’ to represent real-life practice. The frequency of coded events in CYP aged 8–16 years, when otitis externa may present, is a small proportion of the total cohort, but will skew the results. Prescribing for otitis externa may account for the increased use of aminoglycoside topical antibiotics.

It is not possible to ascertain whether antibiotic prescribing was intended for immediate or delayed use. NICE guidelines suggest both are practical treatment options.^9^ Delayed antibiotics can reduce unnecessary use for those whose symptoms will self-resolve. Smith *et al* demonstrated that the majority of CYP with PO in primary care received immediate antibiotics.^1^ It was suggested this was because those who had otorrhoea were more unwell than those without.

The decision to prescribe antibiotics in primary care has been shown to be multifactorial.^18^ Factors are known to include comorbidities (for example, immunosuppression), prior otological surgery, prescribing experience, guidelines, time pressures, and professional and patient preferences.^19^ Within the CPRD the exact reason for a prescription cannot be determined, as the CPRD does not link prescriptions to diagnoses. Consequently, it is not possible to elucidate why prescribers took certain prescribing decisions. The decision-making process is complex, and therefore it was decided it was outside the scope of this study.

Monitoring antimicrobial resistance patterns is essential for antimicrobial stewardship.^9^ It was not possible to collect sufficient microbiological data from the CPRD because of insufficient coding. It is known that approximately 15% of microorganisms found in PO show resistance to amoxicillin.^20^ Co-amoxiclav has lower (5%) resistance.

### Comparison with existing literature

Only one other study, to the authors’ knowledge, has been performed to investigate the incidence of PO, which was performed over a single-year period with 67 000 patients in the Netherlands.^5^ The presentation rate identified in the current study is much lower in comparison (3.15 versus 12.6 per 1000 patient-years).^5^ Reasons for this could include coding variation, study population size, population characteristics, and health-seeking behaviour differences between countries.

A reduction in AOM incidence has been linked with the introduction of the pneumococcal conjugate vaccine.^2^ The gradual reduction of PO incidence in the current study’s cohort may reflect that in PO *Streptococcus pneumoniae* is found in only 6% of microorganisms isolated.^21^

PO places a financial burden on NHS healthcare resources, costing primary care alone around £2 million per year. It is not currently possible, because of coding limitations, to determine the cost to secondary care services, which involves emergency department attendances, specialist appointments, audiology, and surgical management. It is likely there is a sizable personal cost associated with PO. Hollinghurst *et al* identified the cost to caregivers of children with acute cough in the NHS setting.^22^ Translated to PO, the cost to caregivers per year is approximately £608 000 in the UK.

The current results demonstrate the prescribing practices of primary care professionals who are able to prescribe within the NHS, the majority of which are from GPs (82.9% [47 991/57 887]). Private prescriptions are not included. The current results are generalisable to primary care services in the UK. The authors of the current study acknowledge that there is significant antibiotic prescribing variation within primary care.^23^ Factors that can contribute are local deprivation, health professional type, and experience.

Younger age and male sex had a significant effect on the incidence of PO (Table 1). AOM, which occurs before perforation and resultant otorrhoea, is known to particularly affect children in the first 2 years of life.^24^ Males have been previously shown to experience viral infections, such as bronchiolitis, more frequently than females.^25^ The reason for this is uncertain, although some have speculated it could be because of sex hormone effects on T-helper cells.^26^

Children from low socioeconomic backgrounds are known to more frequently experience recurrent ear infections and associated complications.^6^^,^^27^^–^^29^ The data in the current study show the converse of what would be expected in relation to disease incidence relating to socioeconomic background. The reason for this finding requires further research but may suggest there is a barrier or alternative route to healthcare access. This also suggests that the true rate of PO is likely to be much higher than described. In comparison with data from the US, the current data showed no prescribing disparities related to deprivation.^29^

The WHO states that antimicrobial resistance is one of the biggest threats to global public health, data collection on antibiotic use is required, and that evidence-based prescribing should be the standard of care.^10^ In 2010, Smith *et al* showed in a cohort of 38 CYP with acute otorrhoea that 92.1% (35/38) received antibiotics.^1^ The current study’s results demonstrate a slightly reduced rate: 71.9% (57 885/80 454) of CYP with otorrhoea received antibiotics, the majority being oral antibiotics. Of the oral antibiotics prescribed, 79% were amoxicillin in accordance with NICE guidance.^9^ Macrolides were the second and fourth most frequently prescribed oral antibiotic likely used, in accordance with NICE recommendations, for those with a perceived penicillin allergy.^9^ The rate of macrolide prescription (10.5%) mirrors the estimated penicillin allergy prevalence in the UK.^30^ Antibiotic prescribing data collected in this study relate to the initial presentation.

The data in this study show that 19.4% (15 608/80 454) of CYP represent with PO. Previous data has shown that 44% of CYP with PO have at least one further infection within 3 months.^1^ This demonstrates the treatment-resistant nature of PO. Approximately 15% of microorganisms from PO are resistant to amoxicillin and this may correlate with re-attendances.^21^ Resistance to both erythromycin and co-amoxiclav, which are second-line antibiotics outlined by NICE, are 36% and 5%, respectively. The collection of microbial samples in the form of ear swabs is not outlined in NICE guidance for AOM but may play a role to ensure effective antibiotics are administered.

The use of topical antibiotic drops for patients with a tympanic membrane perforation is debated because of the potential ototoxic effects of aminoglycoside antibiotics.^11^^,^^31^ The variation in prescribing practice for topical antibiotics could be related to the lack of guidelines, medication availability, and education. It is interesting that aminoglycoside antibiotics comprise the top three most frequently prescribed topical drops for otorrhoea in primary care (Table 2). Professionals prescribing topical antibiotic preparations may not be aware that they are prescribing aminoglycosides as many drops have numerous drug constituents (for example, Otomize and Sofradex). There is, however, a trend that ciprofloxacin-based topical drops are becoming more favoured over time (Figure 3). Microorganisms causing PO have been shown to have low resistance to ciprofloxacin (4–8%).^22^

### Implications for research and practice

In conclusion, this longitudinal population-based study shows that approximately 41 000 primary care appointments are required for PO each year. Antibiotic treatment most frequently follows NICE recommendations to prescribe oral amoxicillin.

The medical community needs to be cognisant that when otorrhoea is present, especially in younger children, the tympanic membrane is likely to be perforated. This can provide an alternative route for antibiotic delivery in a topical form. Practice is changing from potentially ototoxic aminoglycoside topical antibiotics to ciprofloxacin.

To help improve future primary care research, coding of CYP with PO needs to be distinguished from AOM without otorrhoea. Future research should look to determine the best method of treating PO in primary care, which prevents repeat infective episodes, in turn informing future guidance.

## Data

Electronic health records are, by definition, considered ‘sensitive’ data in the UK by the Data Protection Act and cannot be shared via public deposition because of information governance restriction in place to protect patient confidentiality. Access to individual patient data is not possible. Summary data are made available in the supplementary material.

## References

[1] Smith L, Ewings P, Smith C (2010). Ear discharge in children presenting with acute otitis media: observational study from UK general practice. Br J Gen Pract.

[2] Hu T, Done N, Petigara T (2022). Incidence of acute otitis media in children in the United States before and after the introduction of 7- and 13-valent pneumococcal conjugate vaccines during 1998–2018. BMC Infect Dis.

[3] Liese JG, Silfverdal SA, Giaquinto C (2014). Incidence and clinical presentation of acute otitis media in children aged <6 years in European medical practices. Epidemiol Infect.

[4] Rovers MM, Glasziou P, Appelman CL (2006). Antibiotics for acute otitis media: a meta-analysis with individual patient data. Lancet.

[5] Hullegie S, Schilder AGM, Marchisio P (2021). A strong decline in the incidence of childhood otitis media during the COVID-19 pandemic in the Netherlands. Front Cell Infect Microbiol.

[6] World Health Organization (2004). Chronic suppurative otitis media: burden of illness and management options.

[7] Singer AEA, Abdel-Naby Awad OG, El-Kader RMA (2018). Risk factors of sensorineural hearing loss in patients with unilateral safe chronic suppurative otitis media. Am J Otolaryngol.

[8] Heward E, Dempsey J, Lunn J (2023). Protocol for the Paediatric Otorrhoea Study (POSt): a multi-methods study to understand the burden of paediatric otorrhoea in the UK. BMJ Open.

[9] National Institute for Health and Care Excellence (2022). Otitis media (acute): antimicrobial prescribing NG91.

[10] World Health Organization (2015). Global action plan on antimicrobial resistance.

[11] Phillips JS, Yung MW, Burton MJ (2007). Evidence review and ENT-UK consensus report for the use of aminoglycoside-containing ear drops in the presence of an open middle ear. Clin Otolaryngol.

[12] Medicines & Healthcare products Regulatory Agency (2021). CPRD Aurum June 2021 dataset.

[13] Wolf A, Dedman D, Campbell J (2019). Data resource profile: Clinical Practice Research Datalink (CPRD) Aurum. Int J Epidemiol.

[14] Office for National Statistics (2012). 2011 Census: population and household estimates for small areas in England and Wales, March 2011.

[15] Office for National Statistics (2023). Estimates of the population for the UK, England, Wales, Scotland and Northern Ireland.

[16] Jones K, Weatherly H, Birch S (2022). Unit costs of health and social care 2022.

[17] Paediatric Formulary Committee BNF for Children (BNFC).

[18] Krishnakumar J, Tsopra R (2019). What rationale do GPs use to choose a particular antibiotic for a specific clinical situation?. BMC Fam Pract.

[19] Kasse GE, Humphries J, Cosh SM (2024). Factors contributing to the variation in antibiotic prescribing among primary health care physicians: a systematic review. BMC Prim Care.

[20] Mather MW, Drinnan M, Perry JD (2019). A systematic review and meta-analysis of antimicrobial resistance in paediatric acute otitis media. Int J Pediatr Otorhinolaryngol.

[21] Nawaz S, Smith ME, George R (2023). Changes in antimicrobial resistance in acute otitis media and otitis externa. Clin Otolaryngol.

[22] Hollinghurst S, Gorst C, Fahey T (2008). Measuring the financial burden of acute cough in pre-school children: a cost of illness study. BMC Fam Pract.

[23] Van Staa T, Li Y, Gold N (2022). Comparing antibiotic prescribing between clinicians in UK primary care: an analysis in a cohort study of eight different measures of antibiotic prescribing. BMJ Qual Saf.

[24] Danishyar A, Ashurst JV (2023). Acute otitis media.

[25] Perret JL, Wurzel D, Walters EH (2022). Childhood ‘bronchitis’ and respiratory outcomes in middle-age: a prospective cohort study from age 7 to 53 years. BMJ Open Respir Res.

[26] Muenchhoff M, Goulder PJ (2014). Sex differences in pediatric infectious diseases. J Infect Dis.

[27] Heward E, Saeed H, Bate S (2024). Risk factors associated with the development of chronic suppurative otitis media in children: systematic review and meta-analysis. Clin Otolaryngol.

[28] Vakharia KT, Shapiro NL, Bhattacharyya N (2010). Demographic disparities among children with frequent ear infections in the United States. Laryngoscope.

[29] Qian ZJ, Rehkopf DH (2022). Association between social disadvantage and otitis media treatment in US children with commercial insurance. JAMA Otolaryngol Head Neck Surg.

[30] [No authors] (2017). Penicillin allergy — getting the label right. BMJ.

[31] Roland PS, Rybak L, Hannley M (2004). Animal ototoxicity of topical antibiotics and the relevance to clinical treatment of human subjects. Otolaryngol Head Neck Surg.

